# A focused ethnography of a Child and Adolescent Mental Health Service: factors relevant to the implementation of a depression trial

**DOI:** 10.1186/s13063-017-1982-8

**Published:** 2017-05-25

**Authors:** C. E. W. Kitchen, S. Lewis, P. A. Tiffin, P. R. Welsh, L. Howey, D. Ekers

**Affiliations:** 1 School of Medicine, Pharmacy and Health, Durham University, Durham, UK; 2 Department of Geography, Durham University, Durham, UK; 3 Department of Health Sciences, University of York, York, UK; 4 School of Psychology, Newcastle University, Newcastle upon Tyne, UK; 5 Tees, Esk and Wear Valleys NHS Foundation Trust, Darlington, UK

**Keywords:** Qualitative, Mixed methods, Focused ethnography, Randomised controlled trials, Pragmatic trials, Child and adolescent mental health services

## Abstract

**Background:**

Prior to commencing a randomised controlled trial, we conducted a focused ethnography to ensure that the trial was well suited to the proposed setting.

**Methods:**

A six-month observation of a Child and Adolescent Mental Health Service site in the North-East of England was undertaken to observe the site procedures, staff culture and patient care pathways. During this period, documentary data were collected and interviews were conducted with key informants to provide insight into staff perceptions of the proposed trial. The data were coded using thematic analysis and the resulting themes were verified by a second coder.

**Results:**

Seventeen documents were collected, 158 h of observation and six formal staff interviews were undertaken. Four themes emerged from the data; *non-clinically orientated variation in practice, diagnosis*, *capacity* and *staff economy. Non-clinically orientated variation in practice* occurred when staff decisions were based upon resource availability rather than on clinical judgement. *Diagnosis* demonstrated differing staff confidence in making diagnoses and in the treatment of patients who had received a diagnosis. *Capacity* consisted of the time to attend training and the psychological capacity to consider or incorporate learning into practice. *Staff economy* was characterised by staff changes and shortages. There was significant interaction between the themes, with *staff economy* emerging as a central barrier to research. The results directly informed adaptations to the trial protocol.

**Conclusions:**

An ethnographic approach has provided important insights into the individual, practical and organisational boundaries into which a trial would need to be implemented.

## Background

Randomised controlled trials (RCTs) are generally considered the best way to measure the efficacy of clinical interventions but have been criticised for their lack of applicability to real-world settings [1]. Most RCTs take an explanatory approach to evaluating interventions, whereby the treatment is examined under ideal conditions with carefully defined research participants [2]. Where an intervention has been studied in a highly controlled research setting, it may encounter difficulties when implemented in clinical services. Indeed, the disparity between clinical research and clinical practice has led some commentators to suggest that most research findings bear little relation to a clinician’s everyday practice [3]. This seems plausible in a Child and Adolescent Mental Health Service (CAMHS) environment where the vast majority of clinicians do not regularly conduct research and most researchers are not practicing clinicians. This has been a particular concern for researchers evaluating complex behavioural interventions [1]. A move towards more ‘pragmatic’ trials has attempted to counter these limitations by specifically designing RCTs to mimic the context of the intended setting [1]. A vital step in ensuring that trials are pragmatic is to seek an in-depth understanding of the proposed site and the views of the clinicians who will be involved in the research. In fact, a rigorous approach to trial design that has been informed by the clinical setting, can improve quality and clinical relevance and facilitate strategies that improve the efficacy of the trial itself, as well as limiting the waste of resources within the trial [4, 5].

Despite the identified need for more pragmatic trial designs, there has been a lack of focus on methodologies to guide researchers in this endeavour. The merits of using a qualitative approach alongside a RCT have been well documented and include the ability to question process, understandings and beliefs, not just those relating to outcomes [5–8]. In this paper, we intend to examine the potential afforded by sequencing mixed methods to inform the design of a trial from conception. The approach used in this study was to conduct an ethnography *prior* to, rather than during or after, the RCT to inform the trial design. Surprisingly few mixed-methods studies employ a pre-design component; instead, they tend to focus on using qualitative methods in parallel to the trial or to inform an intervention. A pre-design element enables identification of potential pitfalls in a first draft of a trial protocol, affording the chance to compromise between what is methodologically ideal and what is practically achievable in the clinical setting. This approach may potentially address the recognised mismatch between evidence-based practice (mainly derived from RCTs) and clinical practice [9]. A well-designed trial can ultimately reduce staff burden and ensure a seamless approach to patient care.

## Methods

### Aims

The principle aim of this study was to identify the methodological and organisational factors relevant to the design of a RCT for youth depression in a CAMHS setting. A secondary aim was to describe and document the culture and patient care pathways surrounding depression in this service.

### Setting and participants

Child and Adolescent Mental Health Services in the UK are structured using a four-tier system (see Table 1).

**Table 1:** Description of the Child and Adolescent Mental Health Service (CAMHS) four-tier system of organisation

Tier	Description
Tier 1	Staff in Tier 1 are not mental health specialists (they are GPs, school nurses, etc.). They offer general advice and treatment for less severe mental health problems, mental health promotion and identification of problems early in their development that require more specialist services
Tier 2	Tier 2 are CAMHS specialists working in community and primary care settings who provide assessment and treatment to patients experiencing mental health difficulties, training to practitioners in Tier 1 and outreach to identify severe or complex needs requiring more specialist interventions
Tier 3	Tier 3 are multidisciplinary teams working in the community, providing a specialised service for patients with more severe, complex and/or persistent disorders
Tier 4	Tier 4 provides services for patients with the most serious difficulties and includes highly specialised outpatient teams, day or inpatient units

A case study approach was used and a typical community CAMHS team selected (the proposed study site for the trial). Staff from this service are aligned to one of three providers, all centrally commissioned and based within the same site, consisting of: Tier 2 (targeted services), Tier 3 (specialist services) and Learning Disability (LD) services. The LD team were not included in the ethnography due to the intervention in the subsequent depression trial being unsuitable for LD patients. The team, based in the North-East of England, included a research assistant (*n* = 1), clinical nurse specialists (*n* = 3), a child psychotherapist (*n* = 1), CAMHS clinicians (*n* = 4), an assistant psychologist (*n* = 1), consultant psychiatrists (*n* = 3), an associate specialist (*n* = 1), consultant clinical psychologists (*n* = 3), an associate practitioner (*n* = 1), a community clinician (*n* = 1), clinical psychologists (*n* = 3), an administrator (*n* = 1), team managers (*n* = 2) and a specialist advisory teacher (*n* = 1).

### Study design and data collection methods

An ethnographic approach was used over a six-month period to collect detailed observations, relevant documents and formal interviews. An observational element was selected to closely reflect real-life staff decision-making and compromises. In contrast, document collection and formal interviews were expected to reveal staff knowledge of the official guidance and provide an appreciation of the information available to the team.

This ethnography was ‘focused’ due to entering the field with established research questions, which serves to shorten the length of fieldwork required [9]. The intervention arm of the proposed future RCT had already been identified as Behavioural Activation (BA); a treatment for depression that is designed to encourage patients to spend more time engaged in rewarding activities. This treatment has an established evidence base in adults [10] and recent research has shown promising results in young people with depression [11]. The predefined research questions were ‘*Is there a need for BA therapy in this service?*’, ‘*What would be the barriers to implementation of a RCT of this intervention?*’, ‘*What are the normal care pathways for patients with depression?*’ ‘*How might a BA RCT fit into these existing pathways?*’

A total of 158 h (selected on a purposive basis) of observation was conducted whilst the first named author worked as an assistant psychologist in the CAMHS team (on an unpaid basis). Six formal, semistructured, one-to-one interviews with key stakeholders (see Table 2), each lasting between 16 and 25 min, were also completed. The interview schedules were adapted throughout the duration of the observation and subsequent to each interview to explore emerging concepts. The interviews were audio-taped and transcribed verbatim. Documentary data (*n* = 17) consisted of paper and electronic documents collected whilst onsite; including meeting minutes, emails and a PowerPoint presentation. Methodological triangulation was used to provide more comprehensive insights into each emergent theme [3].

**Table 2:** Pseudonyms and characteristics of the formal interview participants

Pseudonym	Tier	Affiliation
Joan	2	Specialised
Claire	2	Managerial
Leanne	3	Junior
Jackie	3	Psychology/managerial
Judy	3	Managerial
Sarah	3	Psychology

‘junior’ refers to unqualified staff; ‘specialised’ refers to nurses or Primary Mental Health Workers (PMHWs); ‘psychology’ to any qualified professionals aligned to psychology and ‘managerial’ any staff members with significant managerial responsibilities

### Researcher dispositions

As a white female, the ethnographer shared these attributes with the vast majority of staff from the CAMHS team; it has been proposed that shared gender can assist in reducing disparities [12]. The researcher also shared ‘insider status’ as an able-bodied person, with English as a first language and a background in psychology/health. The researcher was an ‘outsider’ due to living outside the immediate area, being younger than most staff and having no employment contract with the NHS trust.

### Analysis

Despite the aforementioned adaptations to the interview schedule, data analysis occurred at the end of the data collection period drawing on experiential knowledge from the field. An inductive approach was taken to analysis to enable meanings to emerge from the data through in-depth examination of all data sets. The ethnographer read the interview transcripts, collated documents and the field diary several times before applying thematic coding to the data, according to the principles of Braun and Clarke [13]. The second researcher independently read through the data sources and a meeting was held between the two researchers. The themes identified by the ethnographer were discussed in detail and verified by the second coder. Conventionally, in most qualitative methodologies, two or more coders would analyse the data. In ethnography, however, the analysis is typically undertaken by a sole researcher because they themselves have become the instrument of interpretation through their in-depth acquired knowledge from the field. In this study, we took a pragmatic stance, blending the two approaches; with the ethnographer leading on the analysis and providing contextualisation acquired from the field to assist the second researcher’s interpretations.

## Results

This study sought to both gain an understanding of the existing CAMHS environment as well as to seek staff views about the possible challenges that a prospective trial would encounter in this context. The enduring impression of the setting was one of a demanding, hectic, service going through a period of intense change. The results are presented under subheadings of the four interlinked themes that emerged from the data; *non-clinically orientated variance in practice, diagnosis, capacity* and *staff economy* (see Table 3)*.* Then the implications for the trial design will be highlighted.

**Table 3:** Four emerging themes

Theme	Description
Non-clinically orientated variance in practice	This theme involves changes to practice described by staff, including the rationale for treatment decisions that are often based upon resource availability rather than clinical need
Diagnosis	This theme consists of staff beliefs and behaviours relating to the treatment and diagnosis of depression
Capacity	This theme consists of the time to engage with research or to attend training and space to psychologically consider or incorporate learning into practice
Staff economy	This theme was characterised by staff changes and shortages

### Non-clinically orientated variance in practice

We were interested in exploring how staff responded to the current guidance surrounding the treatment of young people with depression. Staff demonstrated a good working knowledge of the National Institute for Health and Clinical Excellence (NICE) guidance for treating children and young people with depression [14]. Despite staff awareness of these recommendations, barriers to implementation of this guidance were identified, leading to disparities in patient management.

#### Impact of staff backgrounds

In relation to how staff would currently treat low mood or depression in Tier 2 of the service, Claire explained that due to the diversity of staff within the team, ‘at the moment, it’s a bit of a hit-and-miss scenario’. This variability between different staff members could be explained by the different roles that staff had undertaken prior to joining the CAMHS team and the impact their differing backgrounds had on their approach to treating patients. Joan summarised this view:

‘I think ’cos we do tend to go and do different things. We’re all different backgrounds, PMHWs and we all have different ways of treating people’.

Linked to this, were suggestions that some members of staff struggled to adapt to ever-changing job roles that were a common occurrence in the service.

Staff had assorted training backgrounds and a variety of training opportunities were available to them during the period of the observation. The desire to implement evidence-based practice was highlighted by the team several times; some staff were able to achieve this by attending accredited training programmes through the *Children and Young People’s Improving Access to Psychological Therapies* (IAPT) Service Transformation Programme, whereas others learnt the required clinical skills second-hand from colleagues. Staff expressed a preference for members of the team to have received formal training and highlighted this as a way to improve current practice in terms of treating young people with depression. Staff described these two differing approaches to learning psychotherapy skills, using the Cognitive Behavioural Therapy (CBT) model as an example:

‘We’ve had some CAMHS staff that has been off to do IAPT so they have been trained in CBT… there’s a lot of the staff that’s got that awareness level of CBT so although they can’t use CBT in, in such form they can use approaches of CBT’ [Claire]

‘[To improve current practice: young people need] access across the board to someone who’s CBT-trained and if they’re not getting that then I would kind of be asking what are they getting from a clinician who isn’t CBT-trained? But whether they’ve kind of obviously picked up the principles and haven’t had formal training but they’ve of done kind of workshops and that kind of thing and just from experience because they’ve been in CAMHS for 20/30 years kind of thing. That they’re able to kind of, I suppose they know what they are doing and what’s worked in the past for their clients with depression’ [Leanne]

Staff noted incongruities between the treatments being offered to patients due to the different training that staff may have undertaken. Some staff were concerned about the implications of learning therapeutic skills informally. There were concerns that young people were being treated for depression in Tier 2 but were not receiving evidence-based practice:

‘Depression is, if you don’t deal with it early on, it can reoccur and it, it can be really debilitating for people so we need to tackle it and treat it at this early stage. I don’t have a concern with it being treated in Tier 2, I do have a concern with it being dealt with in Tier 2 by staff who aren’t trained in the treatments for it’ [Jackie]

An informal approach to staff supervision was also observed, during staff discussions informal support and advice was offered about how to best treat patients.

#### Impact of a stretched service

Compounding the variances in training across the CAMHS team, there are a number of tensions within the service that made it difficult to deliver treatment according to recommended NICE guidance. One member of staff [Sarah] reported that young people within Tier 3 had to be assigned to a clinician for treatment ‘based on space rather than need’. Several quotes related to this patient management approach and detailed how young people in the service were assigned to care:

‘[I]t depends on what information we get and it depends on what staff we’ve got. If it’s a young person that they, you know that’s presenting with some depression and we haven’t got a CBT appointment then we’ll put them into another appointment’ [Judy]

‘[Well I suppose it would depend on the clinician individual approach…] adhering to the guidelines really and I suppose young people being assigned to the most appropriate people for their difficulties. I know that doesn’t always happen because of the sheer volume of referrals and lack of capacity with staff that we’ve got*’* [Leanne]

‘[T]he really bad point is really that if we need specific CBT … we then have to put it into ier 3 for them to have that because actually we haven’t got enough CBT practitioners in tier 2 but that doesn’t, that doesn’t mean that the young person should be in tier 3. It’s just, that’s the only way they access CBT’ [Claire]

Staff explained that these treatment decisions were based upon the availability of resources rather than the patient’s clinical need. This can be linked to another identified theme from the data which will be explored further below, which is that of *staff economy*; highlighting staff shortages as one reason why staff are often not able to allocate patients in line with guidance. These difficulties were observed in situ as illustrated in a field note entry: ‘recently the team have been allocating referrals to any clinician (unless a specific treatment such as CBT has been suggested). This means that they are not based on their severity (i.e. more severe cases are not seen by more experienced clinicians currently)’. Management noted ‘it’s better that [patients are] seen than wait…’ highlighting the difficult decisions and compromises that need to be made within a stretched service. Furthermore, staff and patient preferences were often unable to be effected due to the burden of large caseloads, with staff reporting an inability to see patients in a weekly or bi-weekly format which they felt was required for successful treatment, complaining: ‘it’s too long between sessions, need to keep the momentum going and the progress’.

### Diagnosis

Beliefs surrounding diagnoses were divergent and rooted in staff’s professional training backgrounds; paralleling the findings in the *non-clinically orientated variance in practice* theme. The *diagnosis* theme can be illustrated in a vignette involving Jackie, who has a background in psychology, who recounted an interaction with a nurse who had completed an IAPT training course in CBT. The nurse explained that it was one of their core beliefs as a nurse that you do not diagnose and despite moving from an assessment to a treatment-based role, they were reluctant to treat young people who had received a diagnosis. Jackie questioned whether it was possible to treat any patient without identifying the condition being treated. Jackie had advised the nurse to re-evaluate their standpoint in light of their recent psychotherapy training. This vignette demonstrates some of the professional divisions within the service.

Despite these often disparate views that are grounded in staff’s professional affiliations, staff agreed on whose role it was to diagnose depression. Staff in Tier 2 clearly articulated that diagnosis did not fall under their remit:

‘No in Tier 2 we wouldn’t diagnose depression. We would obviously pick up the signs and symptoms from the young person’s presentation and the ROMs [Routine Outcome Measures]. Using tools, but if they wanted a clinical diagnosis of depression then it would have to go to a consultant in specialist CAMHS’ [Claire]

Staff noted that ‘psychology or psychiatry’ were responsible for making diagnoses but also discussed how it was rare to receive a diagnosis in the service. Staff described how a lack of diagnoses had previously caused difficulties implementing a depression pathway within the service. Staff were reluctant to diagnose depression and lacked confidence in decision-making surrounding depression, often relying upon the expertise of specific professionals within the multidisciplinary team:

‘Often people send [patients] to a medic [psychiatrist] because they want the medic to make the decision because they don’t feel confident doing it themselves’ [Sarah]

Lower-grade staff in particular, described a lack of confidence in dealing with young people with a diagnosis of depression. In this context, the finding that some staff tended to separate the symptoms of depression from a clinical diagnosis of depression itself, indicates that whether or not the patient had received what they termed a ‘clinical’ diagnosis had an impact upon staff’s confidence to deal with that patient:

‘When you talk about depression though, do you mean clinical depression, that’s got a diagnosis?’ [Joan]

‘Not if it’s clinical depression, no we wouldn’t, no we would treat low mood but young people will often tell you that they’re depressed without having the diagnosis criteria for depression …’ [Joan]

There was a general consensus from staff about where depression should be treated in the service; low mood or depression without significant self-harm in Tier 2 and depression with self-harm or severe depression in Tier 3. Staff identified ‘early onset’, ‘early stage’ or ‘vague’ depression would sit within Tier 2. Although, some staff felt depression should not be treated within Tier 2 at all:

‘I think the risky ones regarding the depression are the ones that have carried out an act of suicide or significant serious self-harm. We do have a lot that remains in Tier 2 that is low-level self-harm linked to the low mood and depression but we tend to keep them in Tier 2’ [Claire]

‘We wouldn’t normally treat people in Tier 2 who’ve got a diagnosis of clinical depression’ [Joan]

‘I think if we could, I think depression probably shouldn’t sit in Tier 2. I think it should sit in Tier 3. But I think we should have more people in Tier 3 so that if Tier 2 gets a whiff of depression they’re not keeping it, they can pass it straight in’ [Sarah]

Staff suggested that depression would be better placed within Tier 3 and cited that a barrier to this was a lack of staff capacity to provide treatment within Tier 3. This parallels the findings in the *staff economy* theme that will be discussed later.

Treating young people who had received a clinical diagnosis of depression could also be stress-inducing for staff:

‘I have to say that I tend not to keep people who have [depression], particularly if they think they’re depressed. Low mood I might keep them for a little while but I’d tend to pass them on. I’m quite risk averse really. And not being mental health trained…’ [Joan]

‘I think that by highlighting to people that what they are dealing with is depression then it might raise their anxieties a little bit’ [Jackie]

In contrast, some staff felt more confident dealing with depression or self-harm within their roles in Tier 2. Joan commented that there were some members of the Tier 2 team who felt confident treating young people who had received a diagnosis of depression: ‘there are some people who’d hold onto them [patients with a diagnosis] because that’s their background’. Again, this highlights the importance of staff backgrounds. Concerns surrounding staff confidence were expressed at senior levels within the trust. For example, an email from the chief executive noted a lack of staff confidence when it comes to decisions about what information to communicate to the friends and families of patients who are receiving treatment in the trust.

Staff agreed that depression is rarely seen in isolation and often comes with myriad comorbidities. Audit data collected whilst onsite confirmed that 60% of patients experiencing low mood or depression had at least one comorbidity; however, these patients may not have received an official diagnosis.

### Capacity

There were two uniting aspects to this theme; time and psychological capacity. When planning a future pragmatic trial in this service, we wanted to train existing staff to provide the BA intervention. As such, we were interested to explore staff perceptions towards the different types of training that were currently on offer to the team in order to evaluate how the BA training may fit into this context.

#### Psychological capacity

Psychological capacity was the time or ‘headspace’ to mentally consider the training opportunity or incorporate the learning from training courses into practice. When Judy was asked what would influence staff to take up training opportunities, she responded: ‘headspace and capacity I would say is probably one of the main things’. One member of staff used the term headspace to describe their thoughts whilst considering taking part in training:

‘Possibly staff uptake as well. I do think they would really want to do it and find it helpful but it’s gunna be the way that they are approached really because, headspace. If you catch someone on a difficult day and they’re back to back with clients they might not have room in their head to think about something else but if it’s done kind of obviously on a convenient day and just kind of putting it to them in the right way’ [Leanne]

Staff thus introduced the concept of *headspace* spontaneously without prompts from the research team. In this context, this shared terminology was used to describe the need for more thinking space. However, *headspace* is less readily defined than time, as it appeared to mean different things to different people. Claire described a similar concept whereby staff required time to incorporate learning from training into practice:

‘From my experience it’s about support and I think that if … you give that person the time, the opportunity to not only do the training but them to put it into practice and they get the outcomes and feel much better about it. There’s lots of times where people have gone and asked for training, gone off and done the training and come back and not done anything with it’

‘I think given the pressures on the service, the demand of the referrals that are coming in. It’s not always easy to put things into practice’

The team described how they had implemented processes to overcome the difficulties with lack of *headspace* by asking each member of staff who attends training to feed back the learning outcomes to the rest of the team.

#### Time

Staff found it difficult to find the time to attend training events. Despite this, they were keen to explore the possibility of new alternative treatment approaches, especially those that did not involve lengthy training, which can be best illustrated in the following quotes:

‘I think there is always kind of room for more treatments and things. Particularly with it being so easy to kind of train in, so obviously just five days which is a lot easier to squeeze into someone’s diary than doing a diploma for a year or something’ [Leanne]

‘I suppose fitting in the kind of time to do it in their diary. I know it is only five days but with clinicians being booked up quite far in advance it will have to be kind of planned quite early on I think’ [Leanne]

‘There is quite a lot of training around but it’s having the time to do it often’ [Joan]

This cost-benefit analysis by staff led them to make an assessment of their capacity to partake in training, highlighting the significant crossover between the theme of *capacity* and that of *staff economy.* Staff referred to the ‘burden’ of training and noted the competing commitments that they had to balance in their everyday practice. The role of the manager was highlighted as central to alleviating these pressures which can be illustrated in the following example:

‘My concerns are that the staff are overwhelmed and busy and doing all sorts of other things and I’m hoping that the managers have remembered that they are doing this BA training and that they’ve left time and space for it…’ [Jackie]

### Staff economy

**Fig. 1:**
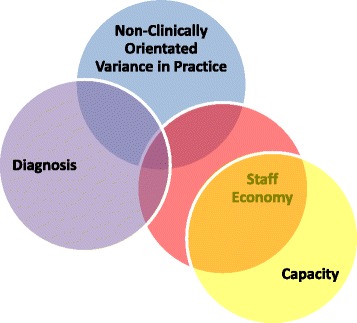
Diagrammatic representation of the themes

This theme was central to the other themes identified (see Fig. 1) and mediated many barriers to trial implementation.

During the period of observation, new working hours were implemented to cover 8 am until 8 pm Monday to Thursday and improve weekend provision. The impact of this change was felt immediately by the ethnographer who noted the lack of staff present onsite due to the new rota and the vacant atmosphere it created. Practical issues with the new working hours were cited: ‘there are also fewer opportunities to see colleagues for case discussions due to longer hours and thinner spread of staff’. Various strategies were observed to overcome these difficulties such as improving staff planning, ensuring that lower-grade staff are utilised for less specialist tasks, introducing more locality-based working to improve efficiency and ensuring that new appointments fill the skills gaps identified within the teams. It was acknowledged that many of these changes were beyond the control of staff themselves and they were encouraged to focus upon their clinical practice:

‘[A] lot is outside our control such as staffing budgets but to remain focussed on what we can control – in the sessions with our clients to be the most effective clinicians we can be’ [Minutes of Meeting]

Staff shortages (i.e. ‘major staffing difficulties reported’) were evident within the team, which were combined with an increase in the number of patients requiring treatment. This was verified in official documents gathered whilst onsite; for example, the minutes of a psychology meeting stated ‘from January psychology staff will be thin on the ground for at least 12 months’ and ‘direct activity has increased’. Again this was echoed during staff interviews:

‘At the moment, the staffing is quite difficult and the numbers are quite difficult’ [Joan]

‘With staff numbers kind of becoming reduced over the previous year and going forward because we do have staff members leaving. Staff members reducing to kind of part-time hours when they’ve previously been full-time’ [Leanne]

It was further demonstrated in the minutes of a team meeting that ‘the group acknowledged it’s hard to say “no” to additional work requests despite no capacity’. This illustrates the links between the *staff economy* and *capacity* themes. Some staff members specified that these shortages were particularly pertinent in the middle job-grade bandings. On the contrary, other team members believed the number of staff was adequate to meet the service need but highlighted that the gap was actually in the staff *capacity* to provide treatment due to staff taking on other commitments. Staff articulated how there has been a change in the profile of the cases that staff see; in that they are now more severe cases. There was a belief that the increased pressures were of a transitory nature and were not permanent with short-term measures often being mentioned: ‘We have got big caseloads at the moment that we don’t normally have’. Despite the enduring theme of staff shortages, during the period of observation there were new staff appointments made.

As in the *capacity* theme, research was felt to be another competing responsibility, along with things like training, which was a source of concern for staff:

‘I guess the only issue [with a trial running in the service] … would be, is if it feels like people are getting taken out of the team again. So if there’s any, where people were “oh I can’t do this or I can’t do that”, people will resent that’ [Sarah]

The value of research was weighed up against the time that staff had available in a stretched service: ‘I guess it’s how you sell it, it’s how much time commitment it involves for people. If it’s not too time-consuming then people are ok with it’. Staff suggested that training should be booked well in advance into clinicians’ diaries and could best be delivered over several weeks rather than being condensed into one week:

‘Just because of the pressures, I have to admit the workers within our team have got a conscience so actually if they gunna be out of the building four days they know that actually when they come back they’ve either got four days of referrals to look at, four days of telephone calls to ring back, four days of appointments to either cancel or rearrange so actually if we do it in blocks of two, two days here and then in a couple of weeks, two days here or one day or whatever … least that would be split nicely in the diaries so they don’t feel that it’s a huge pressure taken out’ [Claire]

When Judy was asked to foresee any difficulties with the proposed research project she responded ‘staff, staffing. The staff to do it’. Staff mentioned the need for support with research projects and highlighted that it was the manager’s role to ‘stop that merry-go-round from going, to actually implement that group because it will be a positive longer term’. Others suggested fostering communication between researchers and managers.

As mentioned earlier, there was a notable interaction between *staff economy* and *non-clinically orientated variance in practice.* Staff felt that they were not able to ‘see kids quick enough’. Meeting minutes demonstrate high numbers of calls from ‘concerned parents’ who noted waiting times and altered appointments being the main issues of concern. In contrast, the team also received many ‘thank you’ messages and positive feedback with Sarah believing that once patients are being treated they are ‘getting a good deal’.

### Implications for the trial design

The results from the four themes identified in the ethnography directly informed the protocol of the planned depression trial (see Table 4). The multifaceted and interlinking themes that were identified from the complex setting has led to the impact upon the trial protocol being equally complex. In an attempt to simplify these implications, they have been presented in tabular form and will be discussed in further detail in the ‘Discussion’ section.

**Table 4:** Illustration of how the key findings from the ethnography led to changes in the trial protocol

Theme	Evidence	Implication for planned trial		
Non-clinically orientated variation in practice and diagnosis	Differing staff backgrounds	Selection of an appropriate control arm	Stratified Randomisation by Tier	Recruitment of a variety of staff from both Tier 2 and Tier 3
	Differing staff training experiences			
Staff economy	Staff turnover/job role fluidity	Recruitment of excess staff		
Non-clinically orientated variation in practice, staff economy and capacity	Lack of staff capacity/staff stress	Five days of training split over several weeks and planned several months in advance	Self-selected sample	
Capacity	Feedback from training to team			Cluster randomisation to reduce treatment contamination
Non-clinically orientated variation in practice	Informal staff supervision			
	Informal learning of therapeutic skills	Group supervision to facilitate learning		
Capacity	Headspace			Five days of training split over several weeks and planned several months in advance
Diagnosis	Lack of staff confidence	Use of a structured interview tool to provide a DSM diagnosis by research team		
	Lack of diagnoses			
Non-clinically orientated variation in practice and staff economy	Speed of patient treatment	Reduce treatment delay and recruitment speed by adding additional study sites		
Diagnosis	Comorbidities	Participant inclusion criteria to include comorbidities		
Diagnosis and staff economy	Depression treated in both Tiers	Recruitment across Tier 2 and Tier 3		Stratified randomisation by Tier
Non-clinically orientated variation in practice	Staff treatment preferences	Perceptions regarding delivery to be explored in qualitative interviews with staff and patients		
Staff economy and capacity	Staff and patient management	Attendance at regular management meeting		Informed recruitment strategy

*DSM Diagnostic and Statistical Manual of Mental Disorders*

## Discussion

This study presents a novel approach to sequencing mixed-methods to inform the design of a RCT in a complex clinical setting. We discuss the implications of the findings for the trial design, as well as the strengths and limitations of this study and methodological approach.

### Implications for the trial design

Staff demonstrated a good working knowledge of the NICE guidelines, which is in contrast to previous research that found a lack of awareness of, and poor familiarity with, clinical practice guidelines amongst physicians [15]. However, there were barriers observed to implementing this guidance in practice which need to be considered in a pragmatic trial.

When designing a RCT, an important factor to consider is the way that patients will be allocated to each treatment option. Randomisation is a process to allocate patients in a way that avoids bias and preserves the internal validity of the study [16]. Due to the small size of the planned trial, it was desirable to achieve roughly equal numbers in each treatment group so a blocked randomisation method had been proposed. Stratification within randomisation is an additional control that can be used when we assume that a variable is a very important predictor of outcome [16]. Stratification ensures there are equal proportions of the variable within each treatment arm. As equal distribution of these variables could not be assumed, due to the observed differences between staff across the two Tiers, the randomisation process could incorporate stratification according to Tier. Another way to attempt to control for influences arising from a complex setting is by the use of a control arm. In a RCT, patients are assigned to either a novel or a control treatment and both groups are followed to compare outcomes from each treatment approach. Understanding the disparities in patient management was useful in selecting an appropriate control arm for the planned trial; ensuring that it would also be meaningful in clinical practice. Therefore, ‘usual care’ was selected as a comparator condition to BA to account for the diversity in staff approaches to treatment. This was as an alternative to other options, such as a CBT control arm, which would not have been representative of the observed clinical practice. Another decision based upon the diversity of staff within the team is that a variety of staff will be recruited from across both Tier 2 and Tier 3 in order to explore which staff may be best suited to delivering the planned intervention. Ethnography has its roots in social anthropology where the common assumption is that all members of communities share cultural beliefs and practices [3]. More recent commentators have speculated that in fact individual members of such groups may hold vastly different views, as highlighted in this study. In light of this, particular attention will be paid to recording staff’s prior training experiences and professional backgrounds and exploring the impact of this on their treatment delivery during the qualitative interviews that will be embedded into the RCT. Correspondingly, research into the implementation of a stepped-care model in primary care found that differing staff views of depression and depression care within a multidisciplinary team combined with a lack of resources hindered the rapid introduction of the model [17]. This mirrors the interaction and overlap between the *diagnosis* and *staff economy* themes and the importance of staff preferences in psychotherapy. Therefore, the feasibility and acceptability of the novel treatment in this context will also be explored during qualitative interviews with staff involved in the trial.

Recruitment of eligible participants is a central focus of any trial. The finding in the *diagnosis* theme that well over half of the young people attending the service with low mood or depression had at least one comorbidity informed the participant inclusion criteria to include comorbidities. This serves to increase the pool of potential participants available for recruitment. When including patients in a depression trial, there is a need to gather accurate standardised information relating to their depression status. While many psychotherapy trials rely upon official diagnoses, our results suggest that a more pragmatic choice would be to work outside of a diagnostic framework; however, this needs to be considered against the detrimental impact that this could have upon the quality of the trial. Patients who did receive diagnoses were only found in Tier 3 which would present practical problems for recruiting eligible patients from Tier 2. If Tier 2 patients needed to be referred to Tier 3 for a diagnosis this could lead to treatment delay, compounding the delays already present in the service due to *staff economy* measures. Despite the lack of diagnoses provided in the service representing a practical barrier to research recruitment, this could be overcome by allocating additional resources. Therefore, the research team will use a structured interview to provide *Diagnostic and Statistical Manual of Mental Disorders* diagnostic criteria. This will ensure that the data collected are of good quality and comparable to other international psychotherapy trials.

Recruitment and retention of staff to trials is another important consideration. During this period of austerity, CAMHS has had to function in an environment where demand frequently exceeds capacity. Our findings highlight these economic restrictions as central to the other themes that have emerged. Previous research has highlighted that when extensive health system restructuring occurs at the same time as a clinical intervention it can lead to uncertainty and a high rate of staff turnover [17]. This may translate to reduced or slower recruitment rates and would cause particular difficulties recruiting and retaining staff who have been trained to deliver trial-based interventions. Recruitment of a greater number of staff than required reduces the individual burden of research and helps to maintain patient recruitment and treatment in the event of staff dropout. The addition of further study sites would allow access to a greater pool of staff to help to account for these observed difficulties, and may improve the patient recruitment rate and speed of recruitment in the trial. Further to this, the *capacity* and *staff economy* themes illustrated practical steps that could be taken to ease the burden of training upon staff members. The five days of BA training will be split over several weeks that will be planned several months in advance in order to ease time pressures and incorporate *headspace*.

In psychotherapy trials, in order to ensure fidelity to the therapeutic approach, supervision is an important factor. The provision of appropriate supervision may fulfil the need for staff to have *headspace* in order to facilitate learning in this context. Learning therapeutic skills second hand, as demonstrated in the *non-clinically orientated variation in practice*, has significant implications for a therapeutic trial. In keeping with this group learning mechanism, group supervision was selected rather than individual supervision. Crucially, the informal supervision, feedback and learning that take place within the team may lead to therapeutic contamination. This suggests that cluster randomisation by site rather than individual randomisation may be best suited to address this limitation and this approach would be possible when there is more than one study site. The *staff economy* and *capacity* themes indicate that close working with team managers is vital to provide forewarning of issues that may impact upon the running of the study, as such regular management meetings will be planned into the protocol of the trial.

### Methodological observations

The novel ‘blended’ approach to data analysis attempted to reconcile an ethnographic methodology with a focused approach. The use of a second coder would not be possible in a traditional, longitudinal ethnography grounded in participant observation where the knowledge and interpretation of the ethnographer has been honed through immersion in the field over many months or years. As such, a second coder would not have the capacity to assist in data analysis. In contrast, this focussed ethnography was heavily reliant upon interview transcripts, offering the opportunity for the analysis to utilise a second coder to verify the resulting themes and interpretations. An approach using multiple coders marries well in a trials context where research teams are the norm, rather than lone researchers. The very different epistemic traditions between clinical research centres (such as trials units) and anthropology have been noted previously and in blending these approaches researchers can encounter difficulties [18]. This study represents the challenges of working at the interface of social sciences and trials and has added to the academic literature about focused ethnographic fieldwork in clinical settings.

Our data emphasise previous arguments stressing the importance of strategies to address local problems when designing RCTs [19]. Many trials rely upon a limited number of site visits to provide sufficient information to inform their trial protocol and the findings from this study illustrate that the breadth and depth of information required could not have been obtained using solely site visits. In contrast, this particular approach to sequencing mixed methods has allowed the research team to anticipate potential pitfalls in a future trial through the more in-depth, longitudinal assessment of the study site. A focused ethnography, therefore, may be an important addition to a trialist’s toolbox at a developmental stage of a mixed-methods project to inform trial design.

### Strengths and limitations

As with any qualitative methodology, the results cannot be assumed to be generalisable to other CAMHS teams. Generalisability is never the aim of any ethnography, although the findings are generalisable in the sense of determining whether ethnographic enquiry can successfully guide RCT design. This approach could be translated to many different settings and could be used in multicentre trials to illuminate local differences between study sites. The strength of this qualitative approach was that it allowed the complex nature of the individual CAMHS site to be characterised and utilised to inform the trial design. An ethnography allowed staff knowledge to be meaningfully contextualised and to consider personal, interpersonal, managerial and societal influences on behaviour. The focused nature of the ethnography may have precluded additional useful data being included in the analysis. The needs of service-users were not incorporated in this study, instead they were addressed with patient and public input following this study. In the early stages of protocol development, any input from young people may have been inappropriate or misguided until the provisional study design was in place.

## Conclusions

We have reflected upon the use of a focused ethnography in a complex setting which has enabled the team to allocate trial resources effectively. We found barriers and facilitators for the implementation of a trial; at individual, group and organisational levels. Despite the opposing epistemic traditions, the findings of this study highlight the importance and utility of ethnography in the pre-design stage of a RCT. The result is a trial that is able to respond to, and can be readily implemented in, a ‘real-world’ clinical setting.

## Abbreviations

CAMHS: Child and Adolescent Mental Health Services
CBT: Cognitive Behavioural Therapy
IAPT: Improving Access to Psychological Therapies
NICE: National Institute of Clinical Excellence
RCT: Randomised Controlled Trial
